# Ketone body 3-hydroxybutyrate elevates cardiac output through peripheral vasorelaxation and enhanced cardiac contractility

**DOI:** 10.1007/s00395-023-01008-y

**Published:** 2023-09-09

**Authors:** Casper Homilius, Jacob Marthinsen Seefeldt, Julie Sørensen Axelsen, Tina Myhre Pedersen, Trine Monberg Sørensen, Roni Nielsen, Henrik Wiggers, Jakob Hansen, Vladimir V. Matchkov, Hans Erik Bøtker, Ebbe Boedtkjer

**Affiliations:** 1 Department of Biomedicine, Aarhus University, Hoegh-Guldbergs Gade 10, 8000 Aarhus, Denmark; 2 Department of Clinical Medicine, Aarhus University, Aarhus, Denmark; 3 Department of Cardiology, Aarhus University Hospital, Aarhus, Denmark; 4 Department of Forensic Medicine, Aarhus University, Aarhus, Denmark

**Keywords:** Ketone bodies, 3-hydroxybutyrate, Contractile function, Vasorelaxation, Metabolism, Enantiomers

## Abstract

The ketone body 3-hydroxybutyrate (3-OHB) increases cardiac output and myocardial perfusion without affecting blood pressure in humans, but the cardiovascular sites of action remain obscure. Here, we test the hypothesis in rats that 3-OHB acts directly on the heart to increase cardiac contractility and directly on blood vessels to lower systemic vascular resistance. We investigate effects of 3-OHB on (a) in vivo hemodynamics using echocardiography and invasive blood pressure measurements, (b) isolated perfused hearts in Langendorff systems, and (c) isolated arteries and veins in isometric myographs. We compare Na-3-OHB to equimolar NaCl added to physiological buffers or injection solutions. At plasma concentrations of 2–4 mM in vivo, 3-OHB increases cardiac output (by 28.3±7.8%), stroke volume (by 22.4±6.0%), left ventricular ejection fraction (by 13.3±4.6%), and arterial dP/dt_max_ (by 31.9±11.2%) and lowers systemic vascular resistance (by 30.6±11.2%) without substantially affecting heart rate or blood pressure. Applied to isolated perfused hearts at 3–10 mM, 3-OHB increases left ventricular developed pressure by up to 26.3±7.4 mmHg and coronary perfusion by up to 20.2±9.5%. Beginning at 1–3 mM, 3-OHB relaxes isolated coronary (EC_50_=12.4 mM), cerebral, femoral, mesenteric, and renal arteries as well as brachial, femoral, and mesenteric veins by up to 60% of pre-contraction within the pathophysiological concentration range. Of the two enantiomers that constitute racemic 3-OHB, D-3-OHB dominates endogenously; but tested separately, the enantiomers induce similar vasorelaxation. We conclude that increased cardiac contractility and generalized systemic vasorelaxation can explain the elevated cardiac output during 3-OHB administration. These actions strengthen the therapeutic rationale for 3-OHB in heart failure management.

**Supplementary Information:**

The online version contains supplementary material available at 10.1007/s00395-023-01008-y.

## Introduction

Increasing cardiac output without detrimentally burdening the heart mechanically and energetically is a key therapeutic aim in patients with cardiovascular disease. Particularly in heart failure—a condition of escalating incidence [18]—we need new therapeutic strategies to raise cardiac contractility and output without elevating afterload.

The ketone body 3-hydroxybutyrate (3-OHB) is produced predominantly by the liver. Plasma concentrations of 3-OHB vary dynamically during physiological and pathophysiological conditions: micromolar concentrations are observed during fed conditions [58], around 2 mM during fasting or exercise [17, 32, 50], 6–8 mM during prolonged starvation [16], and extremes of 10–15 mM during diabetic ketoacidosis [54]. It remains uncertain whether the elevated 3-OHB concentrations in response to SGLT2 inhibitors [51] contribute to the beneficial cardiovascular effects [20].

The myocardium—especially in the failing heart [2, 4]—utilizes ketone bodies to fuel oxidative phosphorylation [5, 38], serving as an alternative to glucose and fatty acids for ATP production. However, 3-OHB can also influence cell functions via actions on the G-protein-coupled hydroxycarboxylic acid receptor HCA2 [29] or via changes in the chemical composition (e.g., pH and [HCO_3_^–^]) of the local environment [8, 9].

Ketogenic diets or ketone ester supplements that raise systemic 3-OHB concentrations apparently improve physical performance [19, 34, 46, 48]. However, the performance-enhancing effects are disputed, and further studies are needed. Infusion of 3-OHB increases cardiac output by up to 40% in humans, including patients suffering from heart failure with reduced ejection fraction (HFrEF) [44]. Despite the increase in cardiac output, blood pressure remains remarkably stable during 3-OHB infusions, suggesting that 3-OHB decreases systemic vascular resistance [44]. A ~75% increase in myocardial blood flow in healthy volunteers receiving 3-OHB supports a vasorelaxant influence, which could be direct or indirect, for instance, due to increased myocardial metabolic rate [23].

3-OHB is a chiral compound with two enantiomers. Endogenous hepatic synthesis is dominated by the D-enantiomer [58], and many clinically available assays measure D-3-OHB exclusively [38]. Differences in elimination half-life and central volume of distribution, however, cause L-3-OHB to accumulate more than D-3-OHB during exogenous administration of racemic mixtures [58] and may suggest distinct metabolic fates and biological activities of the two enantiomers.

In the current study, we explore whether the hemodynamic actions of 3-OHB are explained by direct actions on the heart and vasculature. We test the hypothesis that 3-OHB directly relaxes arteries thereby reducing systemic vascular resistance, lowers venous tone, and enhances cardiac contractility. We further explore whether the systemic vasorelaxation differs between racemic mixtures and the separate enantiomers of 3-OHB.

## Materials and methods

### Animals

Male Sprague Dawley rats (10–15 weeks old) from Taconic Biosciences (Denmark) or Janvier Labs (France) were housed at Aarhus University under a 12-hour light/12-hour dark cycle with *ad libitum* access to chow and water for at least 7 days before experiments.

### Design

We tested hemodynamic effects of 3-OHB in vivo and related them to actions of 3-OHB directly on isolated hearts and blood vessels ex vivo. As detailed below, we investigated effects of 3-OHB on (a) in vivo hemodynamic variables using echocardiography and invasive blood pressure measurements, (b) isolated perfused hearts mounted in Langendorff systems, and (c) isolated arteries (coronary, cerebral, femoral, mesenteric, renal) and veins (femoral, brachial, mesenteric) mounted in isometric wire myographs. We compared effects of racemic mixtures or individual enantiomers of Na-3-OHB to equimolar doses or concentrations of NaCl. To test concentrations of 3-OHB exceeding the pathophysiological range (i.e., >20 mM) and estimate EC_50_ values ex vivo, we substituted Na-3-OHB for NaCl to maintain the osmolarity in the physiological range.

### Echocardiography

We performed transthoracic echocardiography on lightly sedated (3% sevoflurane) rats immobilized in supine position on a heating pad with integrated ECG electrodes. Rectal temperature was measured with a thermal probe and kept constant at 37 °C. Using a 21 MHz rat probe connected to a Vevo 2100 high-frequency ultrasound system (FUJIFILM VisualSonics, Canada), we acquired B-mode images in both short and long axis views 10 minutes after induction of anesthesia (baseline) and again 20 minutes after intraperitoneal administration of racemic Na-3-OHB (1.32 g/kg body weight) or an equimolar dose of NaCl. Na-3-OHB and NaCl were both dissolved at 300 mM in Milli-Q water with pH adjusted to 7.40 at 37 °C. Images were analyzed using Vevo Lab software (FUJIFILM VisualSonics), and left ventricular (LV) volumes calculated using the bullet method [33, 53]: LV volume = 5/6 × LV area × LV length. We report changes in echocardiography parameters from baseline for Na-3-OHB after subtraction of the response to equimolar NaCl.

### Blood pressure measurements

In the same rats evaluated by echocardiography, we one day later measured systemic arterial blood pressure. To prevent coagulation during instrumentation, we administered 50 IU heparin intramuscularly. We placed a solid-state pressure catheter (SPR-869, Millar Instruments, USA) in the right carotid artery of intubated rats that were mechanically ventilated with 3.5% sevoflurane. Rectal temperature was maintained at 37 °C using a heating pad. We measured blood pressure and heart rate 15-20 minutes after intraperitoneal administration of racemic Na-3-OHB (1.32 g/kg body weight) and report differences relative to equimolar NaCl. Each rat received the same compound (Na-3-OHB or NaCl) during echocardiography and blood pressure measurements. We determined dP/dt_max_ from the arterial blood pressure traces using the blood pressure module of the LabChart 8 software (ADInstruments, New Zealand).

### Blood samples and 3-OHB measurements

Tail vein blood samples were drawn immediately after echocardiography and blood pressure measurements. We quantified the combined concentration of D- and L-3-OHB by hydrophilic interaction liquid chromatography tandem mass spectrometry, as previously described [56].

To gauge the baseline blood level of 3-OHB before injection of exogenous 3-OHB, we analyzed a drop of capillary tail blood using the Freestyle Precision Neo point-of-care device (Abbott, USA).

### Solutions

The physiological saline solution (PSS) used for myograph experiments consisted of (in mM [13]): 119 NaCl, 22 NaHCO_3_, 10 HEPES, 1.2 MgSO_4_, 2.82 KCl, 5.5 glucose, 1.18 KH_2_PO_4_, 0.03 EDTA, 1.6 CaCl_2_. The Krebs-Henseleit (KH) solution used for Langendorff experiments consisted of (in mM): 118.5 NaCl, 22 NaHCO_3_, 1.2 MgSO_4_, 4.7 KCl, 11 glucose, 1.2 KH_2_PO_4_, 2.4 CaCl_2_. Solutions with elevated K^+^ were produced by equimolar substitution for Na^+^. For ex vivo experiments, NaCl and the racemic mixture and stereospecific enantiomers of Na-3-OHB were dissolved in PSS or KH buffer heated to 37 °C and aerated with gas mixtures of 5% CO_2_/balance O_2_ (hearts) or 5% CO_2_/balance air (blood vessels) before pH was adjusted to 7.40 using NaOH or HCl.

### Isometric wire myography

Rats were euthanized by exsanguination during deep CO_2_ or sevoflurane anesthesia. Tissues were immediately excised and transferred to ice-cold PSS. Segments of coronary septal arteries, middle cerebral arteries, mesenteric arteries and veins, renal interlobar arteries, caudal femoral arteries and veins, and profound brachial veins were dissected under a stereomicroscope and mounted in PSS-filled wire myograph chambers (DMT 610M and 620M, Denmark) for isometric evaluation [26]. The myograph chambers were bubbled with 5% CO_2_/balance air and heated to 37 °C. Arteries were normalized to 90% of the internal diameter corresponding to a transmural pressure of 100 mmHg [41], whereas veins were normalized to an internal diameter corresponding to a transmural pressure of 20 mmHg [61]. All blood vessels were exposed to a warm-up procedure consisting of five 1-minute long contractions elicited by 60 mM extracellular K^+^. Then, we induced an initial maximal contraction by stimulation with 120 mM extracellular K^+^ and 0.1 μM thromboxane analogue U46619. Vasorelaxation was tested in blood vessels pre-contracted by U46619 to a stable tension equivalent to ~60% of the initial maximal contraction. The response was averaged in the interval of peak vasorelaxation, from 5 to 10 minutes after application of the test compound, and reported relative to the pre-contraction level. Multiple concentrations of Na-3-OHB and NaCl were tested on each blood vessel in alternating order between experiments to control for potential effects of time. We report changes from the pre-contraction level for Na-3-OHB after subtraction of the response to equimolar NaCl. In order to reach a vasomotor response plateau needed for EC_50_ estimation, we performed on coronary septal arteries ex vivo an experimental series where Na-3-OHB was applied in concentrations exceeding the pathophysiological range (i.e., >20 mM). In this series of experiments, we substituted Na-3-OHB for NaCl thereby maintaining osmolarity at physiological level, and we report the vasorelaxations relative to time control experiments. We excluded blood vessels that required more than 1 µM U46619 to reach the desired pre-contraction level. Vasocontractions are illustrated and reported relative to the initial maximal contraction.

### Isolated perfused hearts

Isolated perfused rat hearts were prepared as previously described [53]. In brief, rats were anesthetized by a subcutaneous injection of Hypnorm-Dormicum (fentanyl citrate, 0.158 mg/kg body weight; fluanisone, 0.5 mg/kg body weight; midazolam, 0.5 mg/kg body weight) and mechanically ventilated through a tracheostomy. A bolus of 500 IU heparin for anticoagulation was administered through the femoral vein. Then, the ascending aorta was cannulated via a thoracoabdominal incision and retrogradely perfused at a constant pressure of 80 mmHg with 37 °C KH buffer continuously aerated with 5% CO_2_/balance O_2_. Following transfer to the Langendorff system, a balloon was inserted in the left ventricle through the left atrial appendage and pressurized to 5–8 mmHg to simulate preload. After the hearts had stabilized in KH buffer for 45 minutes, we tested the influence of adding 3 or 10 mM racemic Na-3-OHB or NaCl. We report changes from baseline (measured 10 or 20 minutes after buffer change) for Na-3-OHB after subtraction of the response to equimolar NaCl. We excluded hearts that during stabilization did not fulfill the following inclusion criteria adjusted for rat size [14]: Coronary flow: 10–35 mL/min, arrhythmias: <10 ectopic beats and no sustained ventricular tachycardia or fibrillation, heart rate: 150–400 min^–1^, left ventricular systolic pressure: >120 mmHg.

### Statistics

Normally distributed, continuous data are expressed as mean±SEM. P values smaller than 0.05 were considered statistically significant. The n-values represent the number of rats (i.e., biological replicates) unless otherwise specified. Sample sizes were chosen based on previous experience involving similar methods and tissues. Investigators were not blinded for intervention. Effects of time were eliminated by alternating the order of interventions or performing control and intervention experiments on separate preparations in parallel. We applied two-tailed Student’s *t*-tests to compare a single variable between two groups and used logarithmic transformation or Welch’s correction if variances were unequal between the groups. We evaluated effects of two variables on a third variable using two-way ANOVA followed by Šídák's multiple comparisons tests or, in case of missing values, by mixed-effects analysis. Concentration-response relationships were fitted to sigmoidal functions and the derived parameters were compared using extra sum-of-squares *F*-tests. Data were processed in Microsoft Excel 2016, and statistical analyses performed using GraphPad Prism 10.0.2 software (CA, USA).

## Results

We observed no significant differences in baseline characteristics between the treatment and control groups of rats used for echocardiography and blood pressure measurements (Supplementary Table S1) or between the groups of hearts used for ex vivo evaluation (Supplementary Table S2). Supplementary Table S3 specifies the characteristics of the blood vessels used for ex vivo evaluation.

### 3-OHB increases cardiac output, stroke volume, and ejection fraction

**Fig. 1:**
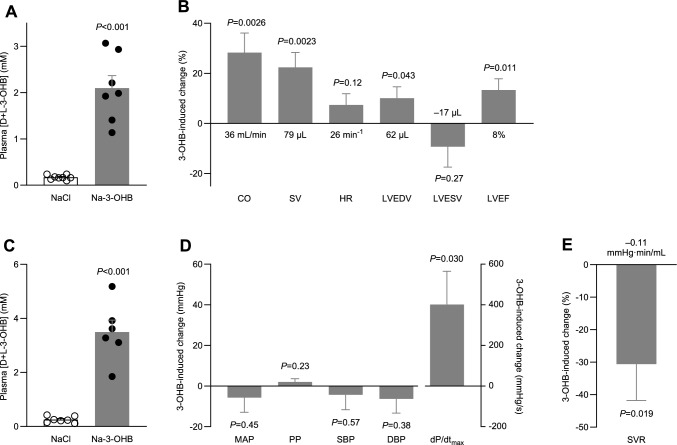
Hemodynamic effects of treatment with 3-OHB. **A**, plasma concentrations of D- and L-3-OHB combined during echocardiography (n = 7–8). **B**, 3-OHB-induced changes in cardiac variables measured in vivo (n = 8). In addition to the relative changes illustrated by the bars, the values at the base of the bars indicate the absolute changes. **C**, plasma concentrations of D- and L-3-OHB combined during invasive blood pressure measurements (n = 6–7). **D**, 3-OHB-induced changes in blood pressure and arterial dP/dt_max_ (n = 6–-7) measured in vivo. **E**, 3-OHB-induced change in systemic vascular resistance (n = 5) measured in vivo. In addition to the relative change in systemic vascular resistance illustrated by the bar, the value at the base of the bar indicates the absolute change. Bars represent mean±SEM. Data in panels **A** and **C** were compared by unpaired two-tailed Student’s *t*-tests with Welch’s correction. In panels **B**, **D**, and **E**, responses to Na-3-OHB and equimolar NaCl were compared by unpaired two-tailed Student’s *t*-tests directly or after logarithmic transformation. Abbreviations: *CO* cardiac output; *DBP* diastolic blood pressure; *dP/dt*_max_ maximal rate of rise of arterial blood pressure; *HR* heart rate; *LVEDV* left ventricular end-diastolic volume; *LVEF* left ventricular ejection fraction; *LVESV* left ventricular end-systolic volume; *MAP* mean arterial pressure; *PP* pulse pressure; *SBP* systolic blood pressure; *SV* stroke volume; *SVR* systemic vascular resistance

For the echocardiographic evaluation, intraperitoneal injection of racemic Na-3-OHB increased plasma 3-OHB concentrations by 1.9±0.3 mM compared to an equimolar dose of NaCl (Fig. 1A). Relative to the osmotic control, 3-OHB raised cardiac output by 28.3±7.8% due to an increase in stroke volume of 22.4±6.0% with no change in heart rate (Fig. 1B). As illustrated in Fig. 1B, the larger stroke volume in response to 3-OHB was associated with an increase in end-diastolic volume of 10.0±4.5% and a similar—yet not significant—relative decrease in end-systolic volume. Together, these effects raised the left ventricular ejection fraction by 13.3±4.6% (Fig. 1B).

### 3-OHB lowers systemic vascular resistance and increases dP/dt_max_ without influencing blood pressure

**Fig. 2:**
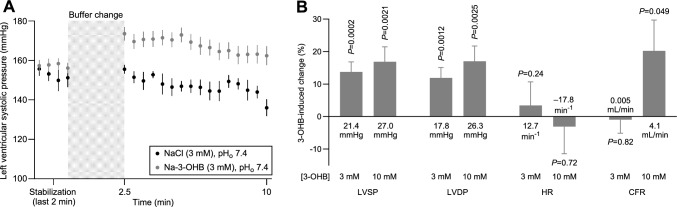
Effects of 3-OHB administration on isolated perfused hearts.** A**, average traces from isolated hearts showing changes in left ventricular systolic pressure during perfusion with Krebs-Henseleit buffer added 3 mM Na-3-OHB or 3 mM extra NaCl (n = 10–12). Data points in panel **A** are also represented in panel **B**. **B**, 3-OHB-induced changes in cardiac parameters measured from isolated perfused hearts ex vivo (n = 9–12) 10 minutes after buffer change. Values after 20 minutes are reported in Supplementary Fig. S1. Bars and symbols represent mean±SEM. In addition to the relative changes illustrated by the bars, the values at the base of the bars indicate the absolute changes. In panel **B**, responses to Na-3-OHB and equimolar NaCl were compared by unpaired two-tailed Student’s *t*-tests. Abbreviations: *CFR* coronary flow rate; *HR* heart rate; *LVDP* left ventricular developed pressure; *LVSP* left ventricular systolic pressure

**Fig. 3:**
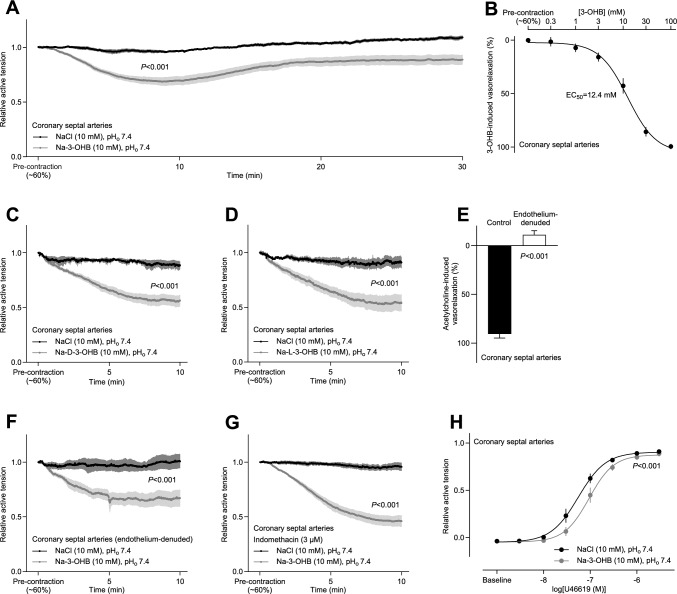
Effects of 3-OHB on isolated coronary resistance arteries. The vasorelaxant responses in panel **A** through **G** are shown relative to a stable U46619-induced pre-contraction. **A**, 3-OHB-induced changes in coronary artery tone (n = 6). **B**, concentration-dependent vasorelaxant responses of coronary arteries to 3-OHB (n = 8). Na-3-OHB was substituted for NaCl and tested by successive full exchanges of the bath solution. The relaxant response was calculated relative to time control experiments where arteries were repeatedly washed to PSS without 3-OHB. In both cases, the concentration of U46619 was kept constant at the pre-contraction level. **C**+**D**, effects of D-3-OHB (**C**, n = 5–8) and L-3-OHB (**D**, n = 4–7) on coronary artery tone. In both cases, Na-3-OHB was compared to equimolar extra NaCl. **E**, assessment of vasorelaxation induced by 5 µM acetylcholine under control conditions (n = 8) and following endothelial denudation of coronary septal arteries (n = 6). **F**+**G**, 3-OHB-induced vasorelaxation in coronary arteries without functional endothelium (**F**, n = 6) and in intact arteries treated with 3 µM of the non-selective cyclooxygenase inhibitor indomethacin (**G**, n = 5). Na-3-OHB was compared to equimolar extra NaCl. **H.** U46619-induced contractions of coronary arteries in presence of Na-3-OHB or equimolar extra NaCl. Vessels were exposed to cumulative stepwise increases in U46619 concentration, and contractions—relative to the initial maximal contraction to 120 mM extracellular K^+^ and 0.1 μM U46619—were fitted to sigmoidal functions (n = 6). Bars and symbols represent mean±SEM. Data in panels **A**, **C**, **D**, **F**, and **G** were compared by repeated-measures two-way ANOVA (interaction) or, in case of missing values, by mixed-effects analysis. In panel **B** and **H**, data were fitted to sigmoidal functions. In panel **E**, data were compared by unpaired two-tailed Student’s *t*-test; in panel **H**, by extra sum-of-squares *F-*test

For the invasive blood pressure measurements, intraperitoneal injection of racemic Na-3-OHB increased plasma 3-OHB concentrations by 3.2±0.4 mM compared to equimolar NaCl injection (Fig. 1C). The recordings revealed no significant effect of 3-OHB on mean arterial blood pressure, pulse pressure, systolic or diastolic blood pressure (Fig. 1D). However, consistent with higher cardiac contractility—suggested by the elevated left ventricular ejection fraction (Fig. 1B)—3-OHB increased the maximal rate of rise of the arterial blood pressure (dP/dt_max_) by 31.9±12.9% (Fig. 1D).

The different plasma concentrations of 3-OHB reached during echocardiography (Fig. 1A) and arterial pressure catheter instrumentation (Fig. 1C) complicate quantitative interpretations. Nevertheless, based on rats with matched recordings of cardiac output and mean arterial blood pressure, we estimate that 3-OHB lowers systemic vascular resistance by 30.6±11.2% (Fig. 1E).

### 3-OHB increases left ventricular contractile function in isolated hearts

To identify the cardiovascular sites of action for 3-OHB, we next evaluated contractile function in isolated perfused hearts mounted in Langendorff systems with constant pre- and afterload (Fig. 2). We compared effects of racemic Na-3-OHB to equimolar NaCl administered to the cardiac perfusate. Relative to the osmotic control, addition of 3–10 mM 3-OHB increased left ventricular systolic pressure by up to 27.0±7.5 mmHg (Fig. 2A, B) and left ventricular developed pressure by up to 26.3±7.5 mmHg (Fig. 2B) within 10 minutes, whereas heart rate was unaffected (Fig. 2B). The effects after 20 minutes were very similar (Supplementary Fig. S1). These findings further support that 3-OHB increases cardiac contractility.

### 3-OHB is a coronary artery vasodilator

We also evaluated effects of 3-OHB on coronary perfusion in isolated perfused hearts mounted in Langendorff systems. Compared to the osmotic control, 10 mM 3-OHB increased coronary flow rate by 20.2±9.5% (Fig. 2B).

To determine whether the raised coronary perfusion in response to 3-OHB is explained by direct vasorelaxation or is secondary to metabolic regulation elicited through effects on cardiomyocytes, we next investigated the function of isolated coronary arteries. The arteries were mounted in wire myographs and pre-contracted with the thromboxane analog U46619 (Fig. 3). 3-OHB caused concentration-dependent vasorelaxation of coronary septal arteries beginning at concentrations above 1 mM and with an EC_50_ value of 12.4 mM (Fig. 3A, B). The vasorelaxant influence of 3-OHB ex vivo peaked after approximately 10 minutes and then reached a plateau that was stable for at least 30 minutes (Fig. 3A).

Racemic 3-OHB consists of two enantiomers, but D-3-OHB dominates endogenous production. To evaluate whether the observations made with racemic 3-OHB are relevant also to conditions with increased endogenous levels—for instance, fasting, ketogenic diets or metabolic disease—we individually tested the two enantiomers. The coronary septal arteries relaxed at similar rate and with similar magnitude when exposed separately to the two enantiomers D-3-OHB (Fig. 3C) and L-3-OHB (Fig. 3D).

In the physiologically relevant concentration range between 2 and 4 mM, the coronary vasorelaxation to 3-OHB (Fig. 3B) was 20–25% of that to 5 µM of the endothelium-dependent vasorelaxant acetylcholine (Fig. 3E). When we elevated 3-OHB concentrations to 30–100 mM—exceeding the pathophysiologically and therapeutically relevant range—vasorelaxation of the coronary arteries was almost complete (Fig. 3B) and similar in magnitude to that induced by 5 µM acetylcholine (Fig. 3E).

We next explored whether the effect of 3-OHB involves changes in endothelium-dependent vasoactive mechanisms. After denudation of the endothelial cell layer—achieved by passing air bubbles through the lumen—the coronary arteries, as expected, did not relax in response to 5 µM acetylcholine (Fig. 3E). However, the endothelial denudation did not restrict the relaxation to 10 mM 3-OHB (Fig. 3F). We also observed no influence on the 3-OHB-induced vasorelaxation when coronary arteries were pre-incubated with 3 µM indomethacin (Fig. 3G), supporting that a cyclooxygenase-dependent prostanoid is not involved.

Finally, we tested whether incubation with 3-OHB influences the sensitivity of septal coronary arteries to a vasoconstrictor agent. Indeed, 10 mM 3-OHB substantially rightward-shifted the concentration-dependency of the vasoconstrictor response to U46619 (Fig. 3H).

Taken together, we conclude that 3-OHB causes endothelium-independent vasorelaxation through a direct action on coronary arteries at concentrations that are both physiologically and pathophysiologically relevant.

### 3-OHB causes vaso- and venorelaxation in several vascular beds

**Fig. 4:**
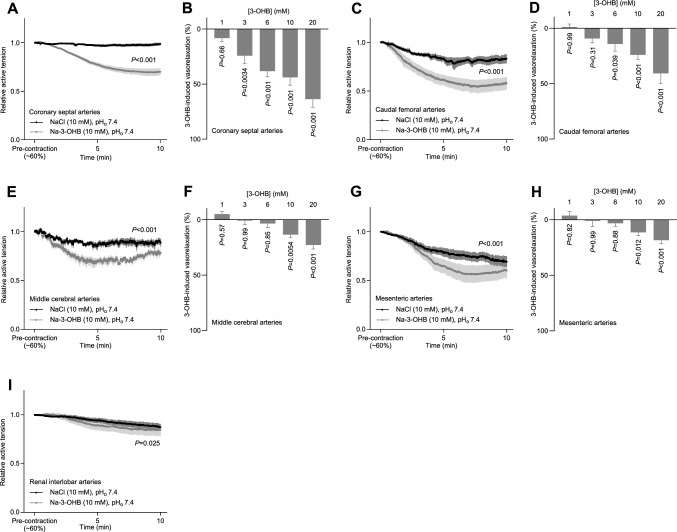
3-OHB-induced changes in vascular tone in different arterial beds. The vasorelaxant responses are shown relative to a stable U46619-induced pre-contraction. **A + C + E + G + I**, 3-OHB-induced changes in coronary septal (**A**, n = 13), caudal femoral (**C**, n = 6–7), middle cerebral (**E**, n = 5), mesenteric (**G**, n = 7), and renal interlobar (**I**, n = 7) artery tone. **B + D + F + H**, concentration-dependent vasorelaxant responses of coronary septal (**B**, n = 7), caudal femoral (**D**, n = 8), middle cerebral (**F**, n = 5), and mesenteric (**H**, n = 7) arteries to 3-OHB. Bars and symbols represent mean±SEM. In all cases, Na-3-OHB was compared to equimolar extra NaCl. Data in panels **A**, **C**, **E**, **G**, and **I** were compared by repeated-measures two-way ANOVA (interaction) or, in case of missing values, mixed-effects analysis. Data in panels **B**, **D**, **F** and **H** were compared by repeated-measures two-way ANOVA followed by Šídák's multiple comparisons tests

We next expanded our evaluation of 3-OHB-induced vasorelaxation to vascular beds other than coronary arteries (Fig. 4A, B), including both arteries and veins. Supplementary Figs. S2 and S3 show average tension traces for each separate concentration of Na-3-OHB and NaCl tested.

**Fig. 5:**
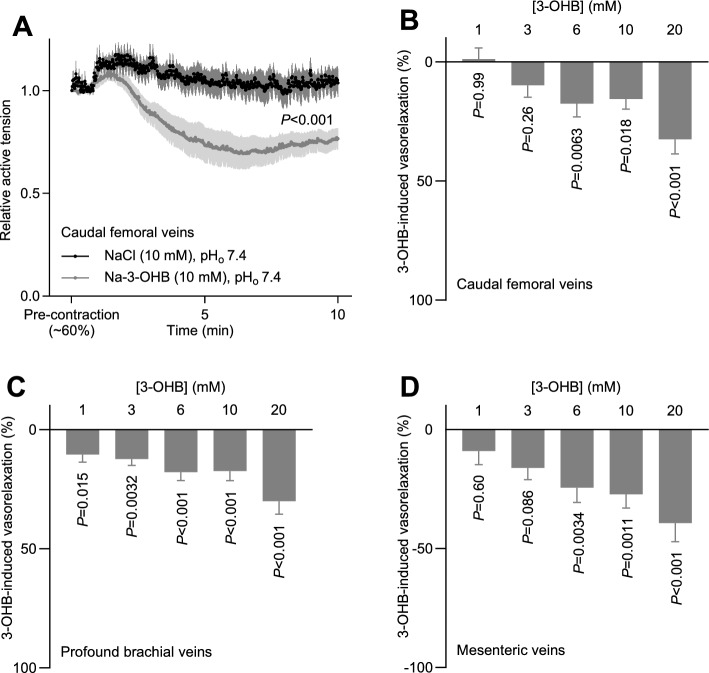
3-OHB-induced changes in peripheral venous tone. The venorelaxant responses are shown relative to a stable U46619-induced pre-contraction. **A**, 3-OHB-induced changes in femoral venous tone (n = 6–7). **B**–**D**, concentration-dependent relaxant responses of caudal femoral (**B**, n = 10), profound brachial (**C**, n = 10), and mesenteric (**D**, n = 8) veins to 3-OHB. Bars and symbols represent mean±SEM. In all cases, Na-3-OHB was compared to equimolar extra NaCl. Data in panel A were compared by mixed-effects analysis; data in panels **B**, **C**, and **D** were compared by repeated-measures two-way ANOVA followed by Šídák's multiple comparisons tests

3-OHB relaxed caudal femoral, middle cerebral, and mesenteric arteries in a concentration-dependent manner, beginning at 6–10 mM (Fig. 4C–H). In contrast, the tone of renal interlobar arteries was only minimally reduced in response to 3-OHB even at a concentration of 10 mM (Fig. 4I). 3-OHB also relaxed caudal femoral, profound brachial, and mesenteric veins in a concentration-dependent manner (Fig. 5A–D). Whereas the profound brachial veins relaxed even in response to 1 mM 3-OHB (Fig. 5C), the caudal femoral and mesenteric veins responded significantly only when 3-OHB concentrations were elevated to 6 mM or higher (Fig. 5B, D).

## Discussion

We show here that the ketone body 3-OHB increases cardiac output (Fig. 1B) without substantially influencing heart rate or blood pressure (Fig. 1D). We further demonstrate that this hemodynamic effect is due to 3-OHB acting (a) directly on the heart to increase cardiac contractility (Figs. 1B, D, and 2B) and (b) directly on the resistance vasculature to cause vasorelaxation (Figs. 3 and 4) and decrease systemic vascular resistance (Fig. 1E).

We reveal the positive inotropic effect of 3-OHB in vivo by the increased left ventricular ejection fraction (Fig. 1B) and the elevated maximal rate of rise of arterial blood pressure (Fig. 1D) and in isolated hearts by the higher left ventricular developed pressure (Fig. 2B) at constant pre- and afterload [55].

Vasorelaxation to 3-OHB ex vivo peaks after 10 minutes and shows a long-lasting plateau (Fig. 3A). In the physiologically relevant concentration range (2–4 mM), the magnitude of coronary vasorelaxation to 3-OHB (Figs. 3B and 4B) is around 25% of that induced by 5 µM of the classical endothelium-dependent vasorelaxant acetylcholine (Fig. 3E). We observe no difference in the vasorelaxant effect of D-3-OHB that dominates endogenous synthesis and L-3-OHB that accumulates preferentially during in vivo administration of racemic mixtures [58] (Fig. 3C, D).

The effective concentration range of 3-OHB and the magnitude of vasorelaxtion vary between vascular beds (Figs. 3 and 4). The particularly pronounced coronary vasorelaxation begins at 1–3 mM, shows an EC_50_ value of 12.4 mM (Fig. 3B), and will redistribute blood flow to the heart. Indeed, we observe a pronounced increase in coronary flow rate when we apply 10 mM 3-OHB to isolated perfused hearts at constant perfusion pressure (Fig. 2B). Through a steepening of the vascular function curve, arterial vasorelaxation in response to 3-OHB (Figs. 3 and 4) will shift blood volume from the arterial to the venous circulation, which may explain our observation that 3-OHB increases left ventricular end-diastolic volume (Fig. 1B). In contrast, the venorelaxation that we document in response to 3-OHB (Fig. 5) can potentially decrease venous return, in agreement with previous data from humans showing reduced central venous pressure when higher doses of 3-OHB are administered [44]. Although we observe no significant effect of 3-OHB on heart rate (Figs. 1B and 2B), it should be noted that our studies are not powered to confirm or exclude the small cardioacceleratory influence (magnitude: 2–7 min^–1^ corresponding to 3–11%) reported in a previous study on humans [44].

Ketone bodies support oxidative metabolism [5, 38] and hold therapeutic promise in patients with severe cardiovascular disease, e.g., heart failure [44], stroke [49], and acute myocardial infarction [63]. The use of inotropic agents in HFrEF patients generally improves hemodynamics and cause symptomatic relief, whereas long-term survival benefits have been difficult to accomplish [1, 45]. It is likely that the increase in oxygen consumption following stimulation of the failing heart is detrimental in patients with limited coronary flow reserve [1]. Hence, the combined positive effect on contractility (Figs. 1B, D and 2B), decrease in systemic vascular resistance (Fig. 1E), and rise in coronary perfusion (Fig. 2B) in response to 3-OHB is therapeutically promising. In contrast to other inotropic agents—e.g., dobutamine—that increase energetic costs of non-mechanical work [59], evidence supports that infusion of 3-OHB in HFrEF patients improves cardiac output without impairing mechanoenergetic efficiency [44]. As 3-OHB fuels oxidative metabolism in cardiomyocytes proportionally to its delivery [39], and to a greater extent in heart failure patients [39], 3-OHB may be of particular metabolic benefit in the failing heart. Other therapies targeting cardiac metabolism show promising results in terms of hemodynamic improvement and survival benefit [27].

Plasma concentrations of ketone bodies vary dynamically: they are low during fed conditions and high during fasting, starvation, and metabolic disorders [16, 17, 32, 50, 54, 58]. The 3-OHB-induced vasorelaxation begins at physiological concentrations (1–3 mM) and continues into the pathophysiological range (Figs. 1, 2, 3, 4). The concentration-dependency of the hemodynamic influences observed in the current study on rats (Fig. 1) match the 3-OHB concentrations that increase cardiac output in humans [44]. Whereas parenteral administration of 3-OHB is impractical for treatment of chronic heart failure, it may be applicable for patients hospitalized for acute heart failure. Ketogenic diets deficient in carbohydrates are in current use for treatment of pharmacotherapy-resistant epilepsy in children [42, 47, 62] and, although controversial, some endurance athletes consume oral ketone ester supplements to improve physical performance [19, 34, 35, 46, 48].

Surprisingly, the two similar intraperitoneal doses of Na-3-OHB administered prior to echocardiography on day 1 (Fig. 1A) and before blood pressure measurements on day 2 (Fig. 1C), respectively, gave rise to rather different plasma concentrations. Although rats after both injections reached a relevant physiological range between 2 and 4 mM 3-OHB, the higher concentrations achieved on the second day of administration suggest that absorption across the peritoneal membrane was accelerated or systemic metabolism delayed during the repeat dosing. Even though we observed no substantial difference in 3-OHB levels between experimental day 1 and 2 in rats receiving NaCl, it is also possible that enhanced lipolysis in response to administration of heparin during blood pressure instrumentation could enhance ketogenesis and contribute to the elevated plasma ketone body concentrations, as previously reported [52]. Based on point-of-care device measurements, the blood concentration of 3-OHB had fully returned to baseline before the second administration of 3-OHB (Supplementary Fig. S4).

Changes in extracellular pH have been known to alter vascular tone for more than a century [21], and the effects of H^+^ in the vascular wall are still being studied intensely [6]. During acidosis, the conjugate base that accumulates alongside H^+^ reflects the metabolic disturbance—e.g., 3-OHB during ketoacidosis—but the functional consequences of these metabolites remain much less studied. In the present study, we isolated effects of 3-OHB from effects of extracellular H^+^ by adjusting all solutions to pH 7.40 before administration. Nonetheless, increasing extracellular 3-OHB concentrations may lead to intracellular acidification by H^+^-linked uptake via monocarboxylate transporters or non-ionic diffusion [24, 25, 40]. Acute large-magnitude intracellular acidification of vascular smooth muscle cells causes vasoconstriction of resistance arteries elicited by an increase in intracellular Ca^2+^-concentration [3, 36]. In contrast, more prolonged intracellular acidification attenuates vascular tone development through a reduction in vascular smooth muscle cell Ca^2+^-sensitivity [7, 10, 11]. In the heart, intracellular acidification lowers contractility [30, 60]. The molecular mechanisms of 3-OHB-induced vasorelaxation and increased contractility need further evaluation. Possible targets include vascular smooth muscle cell ion channels [37] and pH-sensitive enzymes [12]. The influence of 3-OHB on coronary artery tone does not apparently involve cyclooxygenase-dependent prostanoids (Fig. 3G) that are known downstream signals from the 3-OHB receptor HCA2 [22, 29].

Because of chiral specificity of the 3-OHB dehydrogenase, only D-3-OHB is synthesized by hepatic metabolism and utilized under normal conditions [28, 43, 50]. L-3-OHB is either absent or only present at very low concentration in the human circulation [43]. However, exogenous administration of racemic 3-OHB results in augmented plasma levels of L-3-OHB compared to D-3-OHB due to relatively similar half-lives but a much-reduced central volume of distribution [58]. Despite these fundamental differences in metabolism and kinetics, potential therapeutic actions of L- and D-3-OHB can be similar when the signaling activities are without stereoisomeric preference [43]. Our findings show that coronary septal arteries ex vivo respond with equal vasorelaxation in response to the two enantiomers of 3-OHB (Fig. 3C, D), supporting that the described cardiovascular influences are relevant both for exogenously administered and endogenously produced 3-OHB.

## Conclusion

We show here that the ketone body 3-OHB directly elevates cardiac contractility, relaxes systemic resistance arteries, and amplifies cardiac output without substantially influencing heart rate or blood pressure. 3-OHB also causes venorelaxation, which may contribute to the lowering of central venous pressure previously observed with higher dosing regimens.

### Supplementary Information

Below is the link to the electronic supplementary material.

Supplementary file1 A supplementary file containing three supplementary tables and four supplementary figures is available online. (PDF 3385 KB)

## Data Availability

Data will be made available upon reasonable request.
