# Supplementary material for: Ketone body 3-hydroxybutyrate elevates cardiac output through peripheral vasorelaxation and enhanced cardiac contractility
Source: Basic Res Cardiol. 2023 Sep 9;118(1):37. doi: 10.1007/s00395-023-01008-y (PMC10492777; doi:10.1007/s00395-023-01008-y)
Supplement: Supplementary file 1 — Supplementary file1 A supplementary file containing three supplementary tables and four supplementary figures is available online. (PDF 3385 KB) [file 395_2023_1008_MOESM1_ESM.pdf]

Supplementary Material for

Ketone body 3-hydroxybutyrate elevates cardiac output through peripheral vasorelaxation and enhanced cardiac contractility

Casper Homilius[1,*], Jacob M. Seefeldt[2,3,*], Julie S. Axelsen[2,3], Tina M. Pedersen[1], Trine M. Sørensen[1], Roni Nielsen[2,3], Henrik Wiggers[2,3], Jakob Hansen[4], Vladimir V. Matchkov[1], Hans E. Bøtker[2,3], Ebbe Boedtkjer[1]

*These authors contributed equally to this work

[1]Department of Biomedicine, Aarhus University, Aarhus, Denmark

[2]Department of Clinical Medicine, Aarhus University, Aarhus, Denmark

[3]Department of Cardiology, Aarhus University Hospital, Aarhus, Denmark

[4]Department of Forensic Medicine, Aarhus University, Aarhus, Denmark

	Baseline characteristics, echocardiography		
	NaCl (before treatment)	Na-3-OHB (before treatment)	*P*-value
Weight (g)	326±3	317±5	0.21
Cardiac output (mL/min)	144±7	125±11	0.15
Ejection fraction (%)	62.4±1.8	60.7±2.6	0.88
Heart rate (min^{-1})	364±10	350±13	0.40
Stroke volume (µL)	397±19	358±28	0.26
End-diastolic volume (µL)	639±32	585±27	0.22
End-systolic volume (µL)	242±20	227±14	0.57
	**Baseline characteristics, blood pressure measurements**		
	NaCl (before treatment)	Na-3-OHB (before treatment)	*P*-value
Weight (g)	322±4	311±7	0.17

Supplementary Table S1. Baseline values from the *in vivo* echocardiography (n=8) and blood pressure (n=6-7) measurements. The data are mean±SEM and were compared using unpaired two-tailed Student's *t*-tests.

	Baseline characteristics, isolated perfused hearts		
	NaCl (before treatment)	Na-3-OHB (before treatment)	*P*-value
Left ventricular systolic pressure (mmHg)	156.9±3.4	156.6±3.1	0.95
Left ventricular developed pressure (mmHg)	150.2±3.4	150.5±3.1	0.94
Heart rate (min^{-1})	233±10	238±10	0.76
Coronary flow rate (mL/min)	16.2±1.5	15.3±1.0	0.61

Supplementary Table S2. Baseline values recorded at the end of the stabilization period from *ex vivo* experiments on isolated hearts. The table summarizes 21 hearts tested with 3 or 10 mM Na-3-OHB and 19 hearts tested with matching concentrations of NaCl. The data are mean±SEM and were compared using unpaired two-tailed Student's *t*-tests.

	Isolated blood vessel characteristics	
	Lumen diameter (µm)	Initial maximal active tension (N/m)
Arteries		
Coronary septal	309±6	3.23±0.13
Caudal femoral	199±7	4.09±0.26
Middle cerebral	228±8	2.70±0.21
Mesenteric	240±12	7.44±0.30
Renal interlobar	344±18	4.46±0.50
Veins		
Caudal femoral	367±19	1.49±0.10
Profound brachial	616±60	2.44±0.40
Mesenteric	341±39	1.74±0.19

Supplementary Table S3. Characteristics of isolated blood vessels from the *ex vivo* experiments. The arteries and veins were evaluated based on a paired design where each blood vessel was exposed to multiple interventions (e.g., multiple concentrations of Na-3-OHB and NaCl) in alternating order between experiments. Alternatively, two blood vessels from the same animal were tested in parallel (e.g., Na-3-OHB *vs.* NaCl in presence of pharmacological inhibitors). The data are mean±SEM, and n equals number of blood vessels: coronary septal arteries (n=66), caudal femoral arteries (n=20), middle cerebral arteries (n=15), mesenteric arteries (n=20), renal interlobar arteries (n=12), caudal femoral veins (n=23), profound brachial veins (n=10), and mesenteric veins (n=7).

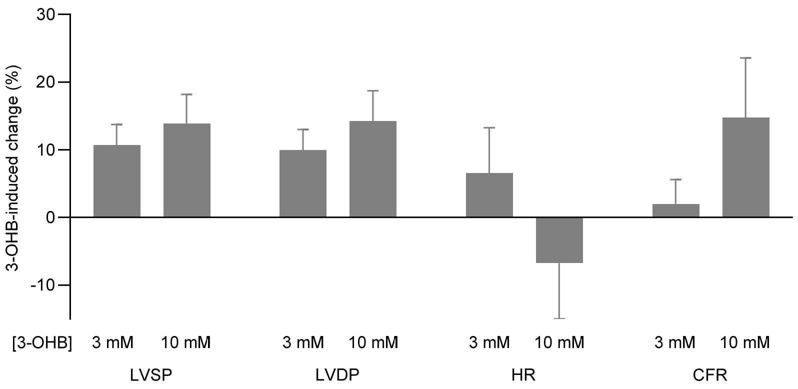

Supplementary Figure S1. 3-OHB-induced changes in cardiac parameters measured from isolated perfused hearts *ex vivo* (n=9-12) 20 minutes after buffer change. Values after 10 minutes are reported in Figure 2. Bars represent mean±SEM. Abbreviations: CFR, coronary flow rate; HR, heart rate; LVDP, left ventricular developed pressure; LVSP, left ventricular systolic pressure.

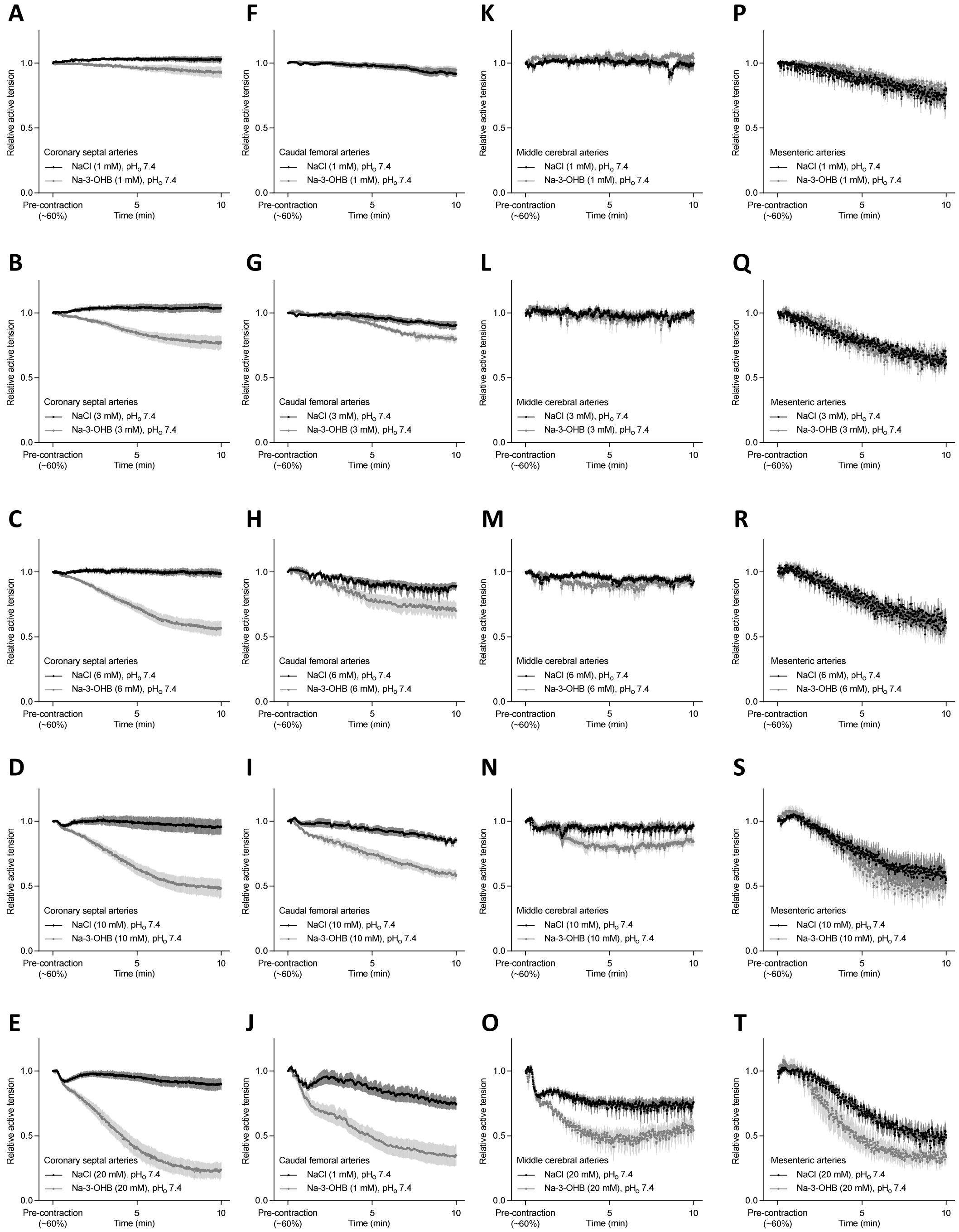

Supplementary Figure S2. Concentration-dependent arterial responses to 3-OHB, illustrated relative to a stable U46619-induced pre-contraction. **A-E**, coronary septal arteries (n=7, summarized data are shown in Figure 3B and 4B). **F-J**, caudal femoral arteries (n=8, summarized data are shown in Figure 4D). **K-O**, middle cerebral arteries (n=5, summarized data are shown in Figure 4F). **P-T**, mesenteric arteries (n=7, summarized data are shown in Figure 4H). The data are presented as mean±SEM.

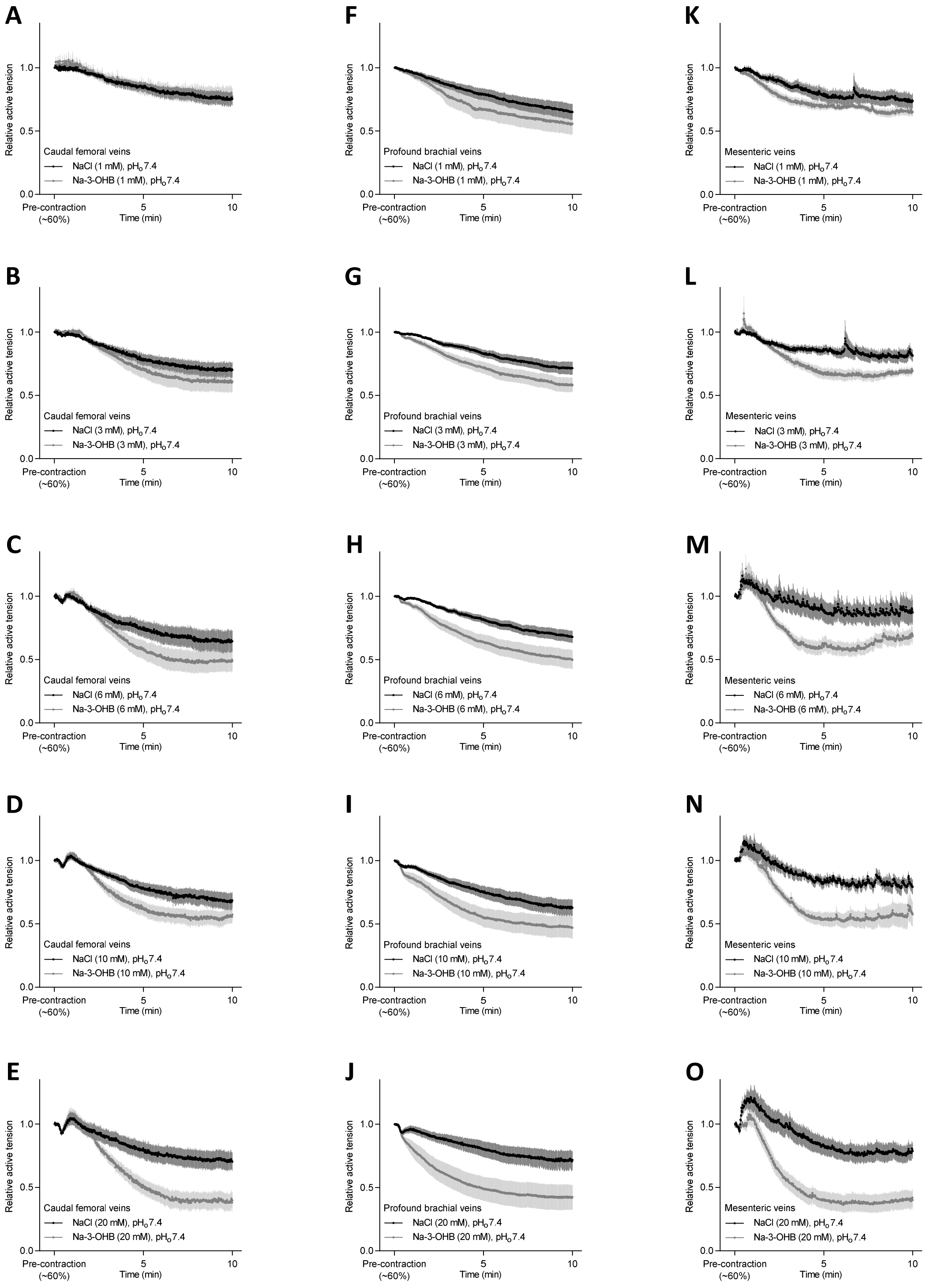

Supplementary Figure S3. Concentration-dependent venous responses to 3-OHB, illustrated relative to a stable U46619-induced pre-contraction. **A-E**, caudal femoral veins (n=10, summarized data are shown in Figure 5B). **F-J**, profound brachial veins (n=10, summarized data are shown in Figure 5C). **K-O**, mesenteric veins (n=8, summarized data are shown in Figure 5D). The data are presented as mean±SEM.

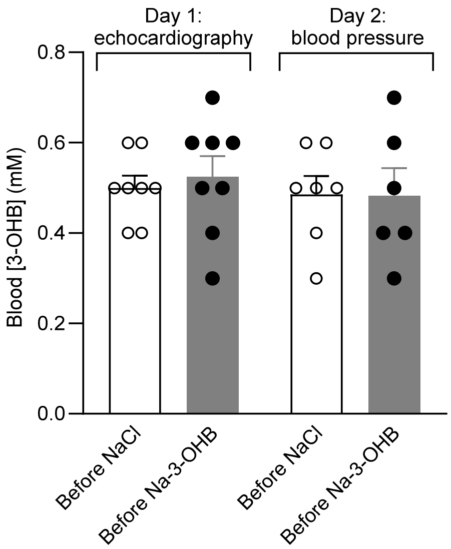

Supplementary Figure S4. Baseline blood concentrations of 3-OHB measured with a point-of-care device prior to administration of exogenous 3-OHB on day 1 (echocardiography) and day 2 (blood pressure) of the study. The bars represent mean±SEM.
